# Targeting heparan sulfate-protein interactions with oligosaccharides and monoclonal antibodies

**DOI:** 10.3389/fmolb.2023.1194293

**Published:** 2023-05-19

**Authors:** Miaomiao Li, Lars C. Pedersen, Ding Xu

**Affiliations:** ^1^ Department of Oral Biology, School of Dental Medicine, University at Buffalo, the State University of New York, Buffalo, NY, United States; ^2^ Genome Integrity and Structural Biology Laboratory, National Institute of Environmental Health Sciences, National Institutes of Health, Research Triangle Park, NC, United States

**Keywords:** heparan sulfate binding proteins, drug target, oligosaccharide, monoclonal antibody, crystallization, specificity

## Abstract

Heparan sulfate-binding proteins (HSBPs) are structurally diverse extracellular and membrane attached proteins that interact with HS under normal physiological conditions. Interactions with HS offer an additional level of control over the localization and function of HSBPs, which enables them to behave in a more refined manner. Because all cell signaling events start at the cell membrane, and cell-cell communication relies on translocation of soluble factors across the extracellular matrix, HS occupies an apical position in cellular signal transduction by interacting with hundreds of growth factors, cytokines, chemokines, enzymes, enzyme inhibitors, receptors and adhesion molecules. These extracellular and membrane proteins can play important roles in physiological and pathological conditions. For most HS-binding proteins, the interaction with HS represents an essential element in regulating their normal physiological functions. Such dependence on HS suggests that manipulating HS-protein interactions could be explored as a therapeutic strategy to selectively antagonize/activate HS-binding proteins. In this review, we will discuss current understanding of the diverse nature of HS-HSBP interactions, and the latest advancements in targeting the HS-binding site of HSBPs using structurally-defined HS oligosaccharides and monoclonal antibodies.

## Introduction

Heparan sulfate (HS) is a sulfated linear polysaccharide universally found at the cell surface and in the extracellular matrix of animal cells. Like other complex carbohydrates, the structure of HS is highly diverse because the biosynthesis of HS is not template-driven. Rather, the final structure of HS is differently regulated in different cell types in a temporally and spatially specific manner. Because of the enormous structural diversity, HS is capable of interacting with hundreds of HS-binding proteins (HSBP) and regulating their biological function in a refined manner. In depth understanding of HS-HSBP interactions will not only help delineate the regulatory mechanisms of HSBPs, it is also essential for development of novel therapeutic agents targeting HSBPs, a good portion of which are suitable drug targets. As the structure details of HS and general principles of HS-HSBP interactions have been well-covered in the literature, we will not discuss them in the current review and refer readers to several excellent reviews on these topics (Esko and Selleck, 2002; Kreuger and Kjellén, 2012; Xu and Esko, 2014; Gómez Toledo et al., 2021).

The purpose of this review is twofold. First, to summarize our current understanding of the specificities of HS-HSBP interactions and the benefits and challenges of determining the structural details of HS-HSBP interaction. Second, to analyze the latest advancement in targeting HS-HSBP interactions using two different pharmacological agents: HS-based molecules and monoclonal antibodies (mAbs). We hope this information will promote the need for better understanding of the structural basis of HS-protein interactions, and clarify the main points investigators need to consider in developing novel pharmacological agents that targets HS-HSBP interactions.

### HS-protein interactions manifest different levels of specificities

The key to understanding HS-protein interactions is to keep an open mind with regard to the specificity of the interaction. HS interacts with hundreds of structurally diverse HSBPs, ranging from tiny chemokines to huge extracellular matrix structural proteins (Xu and Esko, 2014). Due to this extreme structural diversity of HSBPs, the interactions between HS and HSBPs display a huge spectrum of binding affinities, spanning from sub-nanomolar to micromolar (Xu and Esko, 2014). Considering the wide range of structural folds that HS can bind, it is hardly surprising that the specificity of the interactions between HS and HS-binding proteins (HSBPs) can be manifested at many different levels. Broadly speaking, the HSBPs can be categorized into two camps.

1. HSBPs that bind rather promiscuously to a wide range of HS structures, displaying little preference to particular classes of sulfation patterns, as long as the minimum level of sulfation is satisfied.
2. HSBPs that bind in a more stringent manner, usually requires specific sulfation at 2-*O*, 6-*O* or 3-*O* positions.

The second group of HSBPs can be further subdivided into the following two classes based on the degree of stringency with regard to specific modifications.

a. HSBPs display clear preferences for sulfation at specific positions, but these specific modifications are not absolutely required, and without them these HSBPs still bind HS, albeit with lower affinities. Examples of these proteins include neuropilin-1 (Nrp-1) (Thacker et al., 2016), tau (Zhao et al., 2020), FGFR2b (Patel et al., 2014), stabilin (Pempe et al., 2012) and cyclophilin B (Deligny et al., 2010).
b. HSBPs display highly stringent requirement on specific sulfations, and without them the binding becomes nearly nonexistent. Examples of this class include FGF2 (Turnbull et al., 1992), osteoprotegerin (Li et al., 2016), sclerostin (unpublished) (all 2-*O*-sulfation dependent) and antithrombin (3-*O*-sulfation dependent) (Atha et al., 1985; Atha et al., 1987; Richard et al., 2009).

Researchers investigating the binding specificities of HSBPs are now blessed with an array of tools that are widely available. These include CHO cell mutants that are deficient in 2-*O*-sulfation (pgsF) (Bai and Esko, 1996) and N-sulfation (pgsE) (Bame and Esko, 1989), and endothelial cell lines deficient in 2-*O*-sulfation, 6-*O*-sulfation, N-sulfation and 3-*O*-sulfation (Qiu et al., 2018). CHO cell lines have also been created where the role of individual isoforms of 3-*O*-sulfotransferases can be studied (Karlsson et al., 2021). In addition, structurally-defined HS oligosaccharide microarrays have become commercially available (Zong et al., 2017b; Yang et al., 2017; Zhang et al., 2017; Lu et al., 2018), and the oligosaccharides can be purchased for more in-depth studies. These tools will allow researchers to have a comprehensive understanding of the binding specificities of HSBP of interest.

While understanding the specificity is highly important, researchers should proceed with caution when associating specificity with biological significance. An HSBP that binds HS in a less stringent manner does not mean the interaction bears less biological significance than a more stringent HS-HSPB interaction. One should bear in mind that the specificity of a particular interaction has evolved to serve particular biological functions of that HSBP. For some biological functions, such as precise spatial and temporal control of activation/inhibition, a more specific interaction is often preferred. For example, 3-*O*-sulfation is indispensable for activation of antithrombin and for optimum activity of neuropilin-1; and 2-*O*-sulfation is required for the biological activities of FGF and osteoprotegerin (Atha et al., 1985; Atha et al., 1987; Turnbull et al., 1992; Li et al., 2016; Thacker et al., 2016; Li and Xu, 2020). Conversely, for diffusion, gradient formation and cell membrane tethering, a more promiscuous interaction with HS might be more beneficial. Examples in this class include HMGB1, hepatocyte growth factor, interleukin-12, CCL-2, CCL-7 and S100A12. (Catlow et al., 2008; Zong et al., 2017a; Nguyen et al., 2019; Arnold et al., 2020b; Zhang et al., 2020b; Horton et al., 2020).

### Understanding the specificity of HS-protein interactions through structure determination

Understanding the precise molecular details between HS and their targets may be key to unlocking their potential as directed therapeutics. Since HS with varying sulfation patterns can bind a target protein with similar affinities, understanding what functional groups are absolutely required will hopefully allow for generation of HS with high efficacy to the intended target, with fewer off-target interactions. Structures of HSBPs with HS oligomers can provide high resolution atomic detail into the conformation and modifications of the substrate required for high affinity binding. While crystal structures have provided the bulk of complex structures between HS and proteins, recent advances in CryoEM have allowed for structure determination of HS/heparinoids bound to targets (Xie et al., 2017; Dhindwal et al., 2019; Uchikawa et al., 2021; Li et al., 2023). These structures have provided general information on binding locations and potential interactions but so far have lacked the resolution to accurately determine conformational details and specific binding interactions. For this reason, crystallography remains a primary technique for deducing HS/target interactions. However, there are many challenges associated with obtaining HS/protein crystal structures.

Obtaining highly homogenous oligosaccharides with correct length and sulfation pattern that can interact with the target protein should be a prerequisite for any co-crystallization project. Historically HS oligosaccharides were prepared by enzymatic fragmentation of heparin, which could be purified to homogeneous size and utilized for crystallization (Pervin et al., 1995; Toida et al., 1996). However, these purified oligosaccharides are only 70%–90% homogenous because of the structural heterogeneity of heparin, which consists mostly of tri-sulfated (NS, 2S and 6 S) disaccharides, but also contains some less sulfated disaccharides. Nevertheless, heparin-derived oligosaccharides have been used successfully for solving quite a few co-crystal structures (Pervin et al., 1995; Faham et al., 1996; Pellegrini et al., 2000; Schlessinger et al., 2000; Capila et al., 2001; Lietha et al., 2001; Moon et al., 2004; Shao et al., 2006; Li and Huntington, 2008; Fulcher et al., 2017; Vögtle et al., 2019; Griffiths et al., 2021). In addition, heparin mimetics obtained through chemical synthesis have been useful in understanding heparin/HS binding. However, due to the complexity of chemical synthesis, these heparin mimetics were historically limited to short oligosaccharides, such as antithrombin binding pentasaccharide (Jin et al., 1997; McCoy et al., 2003; Johnson et al., 2006). A clear notable exception is the crystal structure of the ternary complex of a 16mer polysaccharide heparin mimetic with thrombin and antithrombin (Li W. et al., 2004).

Recent advances in carbohydrate chemistry and a combination of chemical and enzymatic synthesis, referred to as chemoenzymatic synthesis, allow for the generation of milligram quantities of highly homogenous HS and HS mimics with very specific sequence and sulfation patterns targeting the protein of interest (Mende et al., 2016; Zong et al., 2016; Zhang et al., 2020a; Wang et al., 2020). This has been particularly useful in obtaining structures with the enzymes involved in HS biosynthesis including 2-*O*, 3-*O*, and 6-*O* sulfotransferases, as well as C-5 epimerase (Moon et al., 2012; Patel et al., 2014; Xu et al., 2017b; Wander et al., 2021a; Wander et al., 2021b). Importantly, oligosaccharides synthesized from these techniques are being utilized to generate high-throughput screens. These screens can help determine which types of oligosaccharide structures (in terms of sulfation pattern, uronic acid preference (IdoA vs. GlcA) as well as saccharide length) will be most likely to bind and form crystal complexes (Yang et al., 2017; Chopra et al., 2021).

**FIGURE 1:**
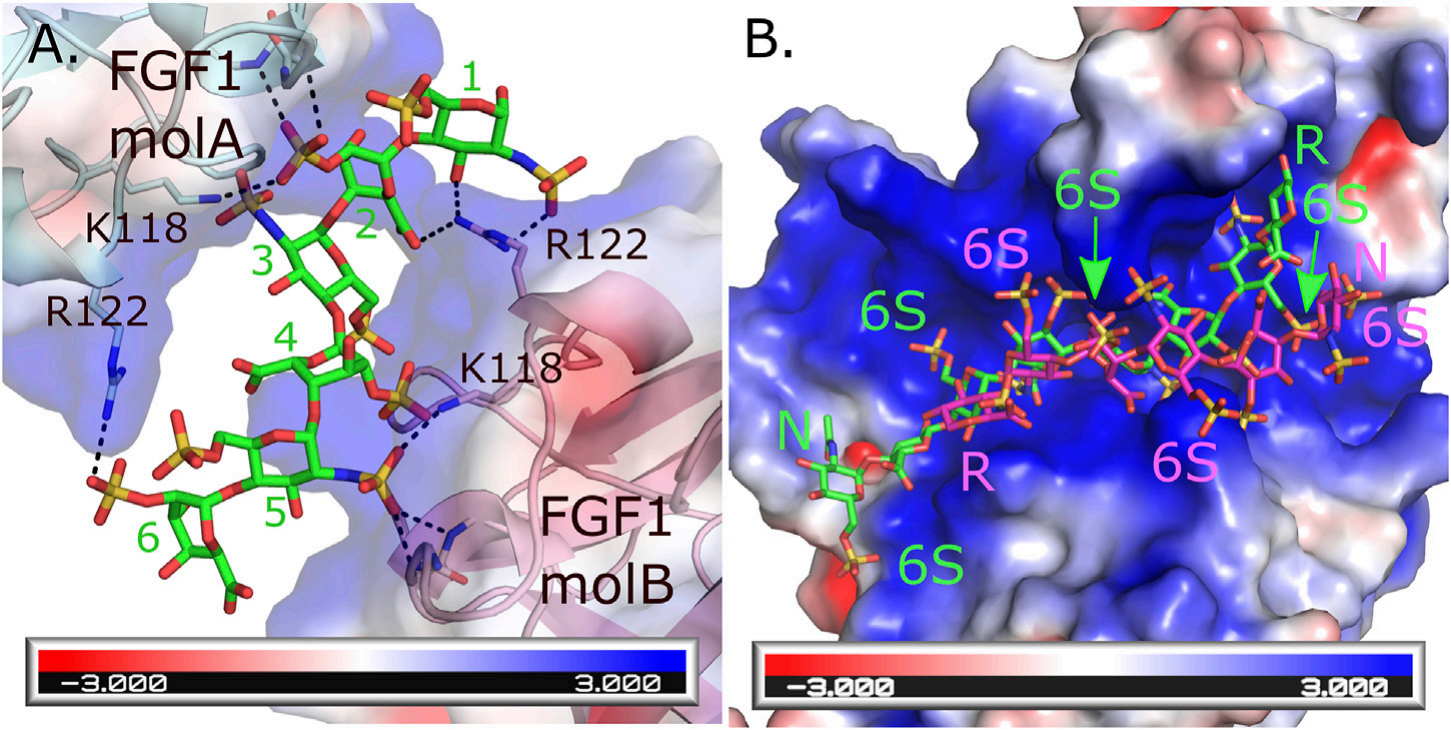
Multiple binding modes for HS to HSBPs. **(A)** Crystal structure of the FGF1 dimer formed through interactions with a hexasaccharide (IdoA2S-GlcNS6S-IdoA2S-GlcNS6S-IdoA2S-GlcNS6S) molecule (green, PDBcode: 2AXM) (DiGabriele et al., 1998). Each molecule of FGF1 interacts differently through similar residues to HS. Hydrogen bonds are shown as dashed black lines. Backbone interactions are from residues K128 and A129 in each monomer. **(B)** Crystal structure of human 3-O-sulfotransferase isoform 3A (3-OST-3A, PDBcode: 6XKG) (Wander et al., 2021b) binding to a 6 S-sulfated octasaccharide (GlcNAc6S-GlcA-GlcNS6S-IdoA2S-GlcNS6S-IdoA2S-GlcNS6S-GlcA-pNP, 8mer) substrate in the catalytically relevant orientation (green). Superimposed onto this structure is the position of the 8mer bound to the other 3-OST-3A molecule in the asymmetric unit where the 8mer is binding in the opposite orientation across the substate binding pocket in a position inconsistent with catalysis (magenta) but capable of inhibiting binding of correctly positioned substrate. This orientation is not seen in the crystal structure when 6-sulfo groups are not present and the enzyme displays greater ability to turn over substrate (Wander et al., 2021b). The HS binding pocket for both FGF1 and 3-OST-3A are highly positively charged. Electrostatic surface potentials are shown for both FGF1 and 3-OST-3A as calculated using the Adaptive Poisson-Boltzmann Solver tool in Pymol (Schrodinger, 2015). Oligosaccharide ends are labeled N and R for non-reducing and reducing, respectively. The 6 S sulfates in the 8mers binding to 3-OST-3A are labeled.

An interesting feature of HS is their ability to bind the same protein in multiple ways. This has been observed in HS’s ability to induce homodimerization of FGF-1 where the HS forms different interactions with similar residues for each of the two monomers (DiGabriele et al., 1998). For monomer A, Arg 122, Lys118 and backbone amides from Lys128 and Ala129 form interactions with the sulfates from saccharides 6 and 2. While these same residues from monomer B form interactions with saccharides 1, 2, 4 and 5 (Figure 1A). In contrast, an octasaccharide substrate of 3-*O*-sulfotransferase isoform 3A (3-OST-3A) has been observed to bind the enzyme in both a catalytically competent orientation, and in a reversed polarity across the active site positioning the acceptor hydroxyl outside the catalytic site (Figure 1B) (Wander et al., 2021b). While intriguing, this heterogeneity in binding can make crystallization of some HS/protein complexes quite challenging. Heterogeneity in binding conformation can result in poorly defined, uninterpretable electron density or interfere with the crystal packing, resulting in absence of crystals. Binding heterogeneity is a property largely dependent on how positively charged residues are arranged in the HS-binding site of the HSBP. When the HS oligosaccharides are highly sulfated, multiple conformations may satisfy the binding requirements of the HSBP. In the case of 3-OST-3A, the nonproductive binding has only been observed when 6-*O*-sulfates are present. These sulfates are not required for binding or activity but can inhibit function (Wander et al., 2021b). Thus, using highly sulfated oligosaccharides could exacerbate the promiscuity problem that is inherent for most HSBPs. To overcome this problem, a logical step would be to simplify the composition of the oligosaccharides to only the minimum requirements of sequence and sulfation. This can be achieved again by using the HS oligosaccharide microarrays to guide the selection of specific HS molecules. In general, a high affinity binding oligosaccharide with fewer sulfates will provide more selectivity compared to a more highly sulfated oligosaccharide. This in theory will translate into fewer allowable binding conformations and a higher chance of obtaining diffraction-quality crystals and interpretable electron density maps. As a cautionary note, it is important for the biologist to download the electron density or CryoEM maps along with the HS-HSBP structures they are studying. Given the potential of multiple binding conformations it is necessary for the individual to critically evaluate for themselves how well the HS model fits the maps and how well the individual protein-HS interactions are determined.

Despite the challenges of determining HSBP-HS complexes, it is essential to further our understanding of the structural details of HSBP-HS interactions to fully realize the therapeutic potential of targeted HS therapies.

### Targeting HS-binding sites with HS-based molecules

Due to the functional dependence of many HSBPs on HS, targeting HS-HSBP interactions represents a viable pharmacological approach to manipulate the functions of HSBPs. To most people outside the field, it must be surprising to learn that HSPBs are deeply involved in most human diseases. For instance, many key players in cancer, including VEGF (Krilleke et al., 2009), Nrp-1 (Vander Kooi et al., 2007), FGFs (Goodger et al., 2008; Belov and Mohammadi, 2013), FGFRs (Loo et al., 2001; Loo and Salmivirta, 2002) and April (Hendriks et al., 2005; Tsiantoulas et al., 2021), are HSBPs. Also, major mediators of inflammation, including all chemokines, cytokines such as IL-6 (Mummery and Rider, 2000), IL-12 (Nguyen et al., 2019) IFN-gamma (Lortat-Jacob and Grimaud, 1991), IFN-beta (Gordts et al., 2014), HMGB1 (Xu et al., 2011) and S100 proteins (Zhang et al., 2020b), are also HSBPs. In bone disease, key mediators such as osteoprotegerin (Li et al., 2016), sclerostin (Veverka et al., 2009), Cathepsin K (Li Z. et al., 2004; Wilson et al., 2009) and BMPs (Jiao et al., 2007; Kim et al., 2021) are HSBPs. In cardiovascular disease, key HSBPs include serine proteases (Carter et al., 2005), serpins (Rein et al., 2011), apolipoproteins (Ji et al., 1993; Gonzales et al., 2013) and RAGE (Xu et al., 2013). In amyloid diseases, common amyloid proteins such Tau (Zhao et al., 2020) and amyloid precursor–like proteins (Gralle et al., 2009; Xue et al., 2011) are both HSBPs.

Heparin has been used as a highly effective anticoagulant in the clinical for more than 80 years now (Linhardt, 2003). Heparin is a highly sulfated version of HS made primarily by connective mast cells and is shorter in length compared to HS made by other types of cells. (Xu and Esko, 2014). The potent anticoagulant activity of heparin stems from its phenomenal ability to promote the antithrombin-thrombin interaction by binding simultaneously to both molecules, which efficiently shuts down the coagulation cascade (Olson and Björk, 1991; Li W. et al., 2004). In addition, binding of heparin to antithrombin requires a specific pentasaccharide structure that contains a 3-O-sulfated glucosamine. Heparin binding induces a dramatic conformational change of antithrombin, which is essential for it to inhibit thrombin and Factor Xa with maximum efficiency (Olson et al., 1992; Jin et al., 1997). The success of heparin is no less than a miracle if one considers how many proteins heparin would bind once injected. As a highly sulfated form of HS, in principle heparin can bind all HSBPs it encounters in the body and alter their forms and functions to different degrees. Many of these alterations could be harmful to the patients. The fact that heparin works at all is due to three main factors. First, it works as an agonist instead of an antagonist for the antithrombin-thrombin interaction (Björk and Lindahl, 1982; Olson and Björk, 1991). Second, the speed of activation is rapid, i.e., within minutes, and the reaction happens in the blood without requiring tissue penetration. Third, heparin is cleared rapidly from the body within a couple of hours (McAvoy, 1979), which ensures that any negative effects it initiates are short-lived.

Despite the great success of heparin, and the large number of HSBPs playing key roles in various pathophysiology, no other therapeutics directly targeting HS-HSBP interactions has entered clinical use. The lack of success in exploring broader therapeutical use of HS and HS-like molecules can be attributed to several factors.

1. Unlike the use of heparin in anticoagulation as a rapid-acting agonist, in most settings the intention is to use HS as an antagonist, which often requires sustained exposure (Lindahl, 2007; Weiss et al., 2017).
2. HS-based molecules are difficult to deliver into tissues. The fast clearance of heparin from plasma has little impact on its use as an anticoagulant, but it is disadvantageous if sustained exposure to an HS-based drug is desired (McAvoy, 1979; Xu et al., 2017a)
3. A more target-based approach requires a structurally defined HS oligosaccharide. The technique of synthesizing such oligosaccharides in large quantity has only recently become available (Mende et al., 2016; Zong et al., 2016; Zhang et al., 2020a; Wang et al., 2020).

These challenges largely explain why additional success with HS-based therapy has yet to be realized despite the potential of such a strategy. In the past several years however, significant progress has been made in exploring the application of structurally defined HS oligosaccharides in inflammatory diseases. The anti-inflammatory effect of heparin has been known for over 30 years. In a sense, it is logical to expect the next breakthrough in HS-based therapy will occur in this arena because of several properties unique to inflammation, especially acute inflammation. First, acute inflammation involves rapid action of inflammatory mediators (cytokines and chemokines) (Sokol and Luster, 2015; Hsu et al., 2022). Inhibition of these acute phase factors does not usually require prolonged exposure to HS-based molecules, which minimizes the problem of the fast clearance rate associated with HS-based molecules (McAvoy, 1979; Arnold et al., 2022). Second, a large number of molecular events in acute inflammation occur in the blood, which greatly increases the chance of success for HS-based therapeutics. In addition, inflammation often involves tissue necrosis and vascular leakage (Pober and Sessa, 2014; Yang et al., 2015), which indirectly promotes tissue penetration of HS-based therapeutics.

In a recent report, a structurally defined 18-mer HS oligosaccharide was found to provide significant hepatoprotective benefit for acetaminophen (APAP)-induced acute liver injury in a murine model (Arnold et al., 2020b). In APAP overdose, necrotic hepatocytes release high mobility group box 1 (HMGB1), an unconventional chemokine that drives neutrophil infiltration into the liver and severely amplifies liver injury. The HS 18-mer functions by binding to HMGB1 and effectively inhibits HMGB1-dependent neutrophil infiltration, thereby preventing further inflammatory damage to the healthy tissue. The authors found that subcutaneous injection of 18-mer as late as 6 h after overdose still provided a significant benefit, which represents a significant improvement over current treatment using N-acetyl cysteine (Nac). It is important to note the 18-mer used in the study is undersulfated compared to heparin, only bearing N-sulfation and 2-*O*-sulfation. What’s interesting is that another more highly sulfated anticoagulant 18-mer (18mer-AXa), despite having 3 times better affinity to HMGB1 than the undersulfated 18-mer, did not provide any protection against APAP overdose. It is suggested the 18mer-AXa did not work because it binds more HSBPs than the undersulfated 18-mer (in particular antithrombin), which negates the beneficial effects afforded by HMGB1 inhibition (Arnold et al., 2020b).

The same 18-mer has also been tested in a murine sepsis model and was found to greatly protect mice from septic shock (Liao et al., 2023). In this disease setting however, the 18mer works through very different mechanisms. The 18-mer functions by neutralizing the cytotoxicity of histone H3, and by liberating apoA1 from high density lipoprotein particles, which in turn sequesters toxic lipopolysaccharide molecules that are abundant during sepsis and promotes their clearance. In another study, the same group also tested the effectiveness of various 12-mer HS oligosaccharides in ischemia/reperfusion liver injury (Arnold et al., 2020a). Unexpectedly, they found that in order to provide benefit to this type of livery injury, both anti-coagulant and anti-inflammatory properties of the oligosaccharide are required. In sharp contrast to the above-described APAP-induced liver injury, in this setting the 12-mer that binds only HMGB1 did not provide protection.

From these reports, one can clearly see the great potential of structurally defined HS oligosaccharide in treating various inflammatory diseases. They also highlighted the importance of testing and comparing the effectiveness of multiple HS oligosaccharides with different sulfation patterns in a given disease model. Because HS oligosaccharides can interact with a broad array of inflammatory mediators, it is nearly impossible to predict what type of structure will provide better protection. It is also quite possible that an undersulfated oligosaccharide performs better than its more highly sulfated counterparts (as in the case of APAP-induced liver injury), because the undersulfated oligosaccharides tend to be more selective than the latter.

### Targeting HS-binding sites with mAbs

HS-HSBP interaction involves binding of HS to a specific HS-binding site of HSBP. The approach using HS-based molecules aims to compete with endogenous HS proteoglycans (HSPGs) and therefore disrupt the normal biological function of HSBPs. An alternative approach to inhibit the interaction would be to block the HS-binding sites of HSBPs. In theory, this can be done with either small molecules inhibitors or with mAbs that specifically target the HS-binding site of an HSBP. But due to the nature of HS-binding sites, which often occupies a large, positively charged surface area (300–600 Å^2^) (Xu and Esko, 2014), it will be extremely difficult to identify small molecules with desirable pharmacological properties that block HS-binding sites. In contrast, mAbs would be an intriguing choice for blocking the HS-binding site for the following reasons.

1. HSBPs are commonly either secreted or transmembrane proteins. These proteins are excellent targets for mAb therapy, since the vast majority of mAbs on the market target these two classes of proteins (Lu et al., 2020).
2. HS-binding sites are completely surface exposed and therefore expected to be highly immunogenic. This property greatly increases the chance of obtaining mAbs with desirable epitopes.
3. It is relatively straightforward to develop mAbs with binding affinities in the sub-nM range, which is highly likely to give mAbs a competitive advantage over HS in binding to the HS-binding site.
4. Compared to HS oligosaccharides-based approach, where one has little control over how many HSBPs it binds *in vivo*; mAb-based approach is a fully targeted approach, where only one particular HS-HSBP interaction is blocked.
5. In contrast to HS oligosaccharides, which have half-lives of a few hours in the body (Arnold et al., 2022), the half-lives of mAbs are usually between 1 and 4 weeks (Mankarious et al., 1988; Keizer et al., 2010). This astounding difference suggests that mAbs would be a much better choice in tackling chronic diseases.

To validate this new strategy of targeting HS-binding site with mAbs, development of mAbs that inhibit the interaction between HS and RAGE is underway. RAGE is a single-transmembrane receptor involved in numerous inflammatory diseases and has been actively pursued as a drug target (Hudson and Lippman, 2018). HS-RAGE interactions are absolutely required for RAGE signaling because HS-dependent RAGE oligomerization is an essential component in RAGE signaling (Xu et al., 2013). By blocking the HS-binding site of RAGE with a mAb, it should be possible to inhibit RAGE signaling by preventing RAGE oligomerization. In a recent report, a mAb that targets the HS-binding site of RAGE has been reported (Li et al., 2022). The epitope of this mAb covers 2 out of 7 basic residues that comprises the HS-binding site of RAGE. Biochemical analysis found that a partial blockade of the HS-binding site is sufficient to greatly reduce HS-binding capacity of RAGE and completely block RAGE-oligomerization. As expected, this mAb displayed excellent biological activity and effectively blocks RAGE signaling in a number of pathological settings where RAGE plays an essential role, including osteoclastogenesis and APAP-induced acute liver injury (Li et al., 2022). Our success strongly supports that targeting the HS-binding site of HSBP with a mAb represents a valid pharmacological approach.

This approach has also been justified in another study where the researchers aimed to block the interaction between HS and proprotein convertase subtilisin/kexin type 9 (PCSK9), an interaction that is essential for inducing low-density lipoprotein receptor (LDLR) degradation (Gustafsen et al., 2017). In this study, several mAbs were generated that specifically recognize part of the HSPG-binding surface in PCSK9. These mAbs effectively blocked HS-PCSK9 interaction and increased the uptake of LDL in HepG2 cells. Importantly, they also found that potency of these mAbs in promoting LDL uptake was similar to evolocumab, a recently FDA-approved mAb targeting the LDLR-binding surface of PCSK9 (Sabatine et al., 2017).

**FIGURE 2:**
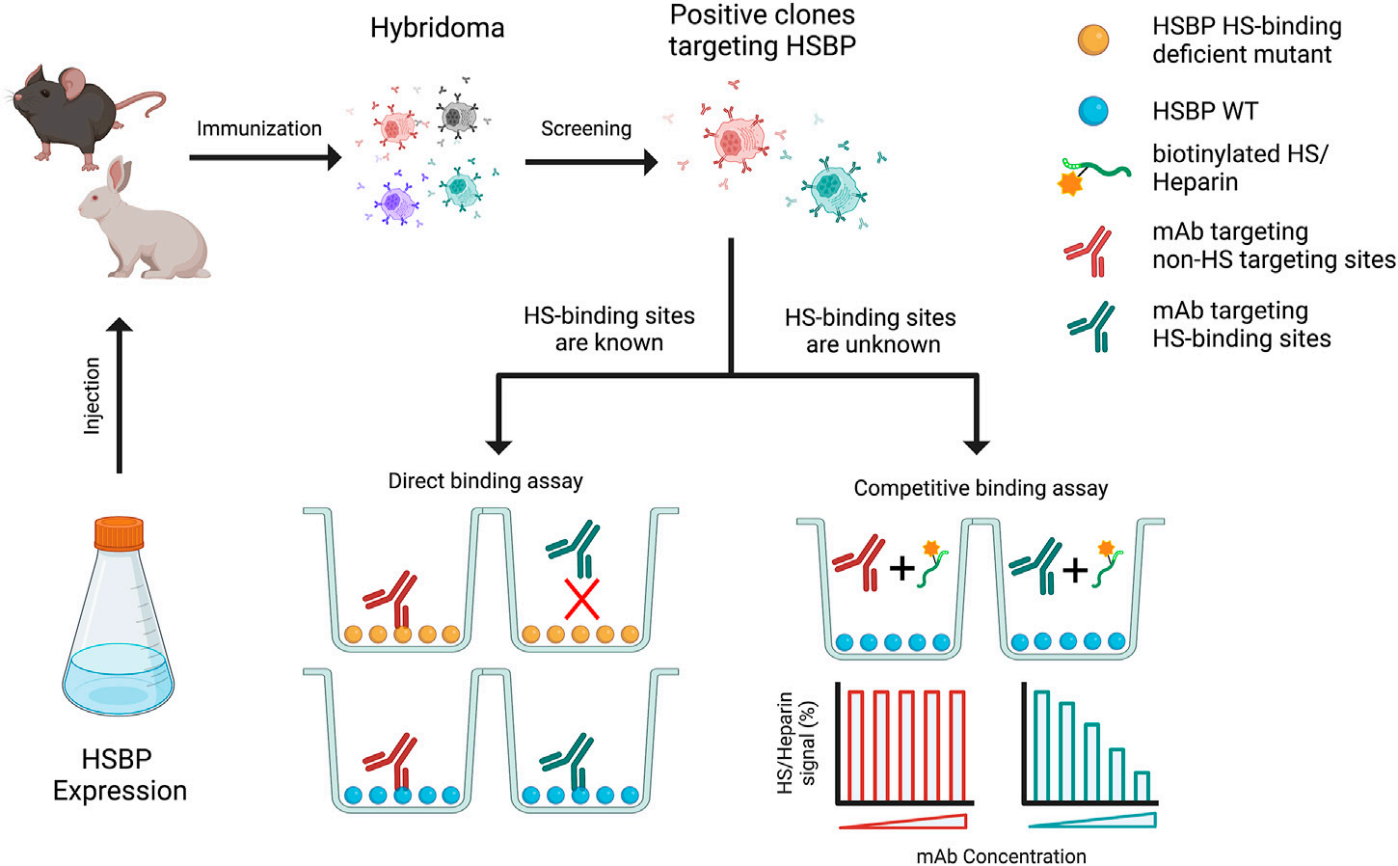
Schematic diagram of generation of monoclonal antibody (mAb) that targets HS binding sites. Purified HSPB was used to immunize the animals, which will be further subjected to generate hybridoma. The selected positive hybridoma clones that target HSBP will be further screened using two strategies, based on whether the heparan sulfate binding sites are known.

The process of purposely developing mAb towards HS-binding sites can be achieved in several ways. If the HS-binding site has already been characterized, mAbs that targets the HS-binding site can be identified straightforwardly by epitope mapping using HS-binding site mutants. This is most conveniently done with a simple, relatively high-throughput, ELISA-based binding assay. Both studies described above used the mutant-based epitope mapping method (Gustafsen et al., 2017; Li et al., 2022). In the case that the HS-binding site is not known, identification of mAbs targeting HS-binding site would require different types of binding assays. The simplest format would be checking whether co-incubation of target protein and mAbs inhibits binding of HS to the target protein. For example, the target protein can be immobilized on a 96-well plate, and the binding of biotinylated-heparin (or HS oligosaccharides) to the target protein can be measured with or without mAbs (Figure 2).

It is important to note that there are also other reports on mAbs that function through blocking the HS-binding site. These mAbs were originally identified through functional screening, but later were found to target HS-binding sites. Interestingly, many such examples are mAbs that inhibit viral infection. In one study, three neutralizing mAbs towards eastern equine encephalitis virus (EEEV) were found to interact with HS binding residues of its envelope protein (Hasan et al., 2018). In another study, two mAbs with neutralizing activity towards human metapneumovirus (hMPV) were also shown to partially blocks the HS binding site of hMPV fusion protein (Huang et al., 2021). These findings suggest that developing mAbs that target the HS-binding site of viral proteins might be a good general therapeutic strategy because many viruses, including HSV (WuDunn and Spear, 1989; Gandy et al., 2022), Dengue virus (Dalrymple and Mackow, 2011) and SARS-CoV-2 (Clausen et al., 2020), utilize HS as the initial attachment receptor.

## Conclusion

Given the roles HS plays in so many physiological and pathophysiological processes, the potential is great for new therapies that target HS-HSBP interactions, whether as agonist or antagonist. Advances in synthesis of HS and HS-like oligosaccharides, and a deeper understanding of the structural basis of these interactions should go a long way in accelerating novel treatments for many diseases. In addition, targeting the HS-binding site with mAbs has emerged as a novel strategy of manipulating HS-HSPB interactions. This strategy should be widely applicable because HS-binding sites are always surface-exposed and should in general have good immunogenicity. These two complementary methods will certainly provide researchers with flexibilities in developing novel therapies that targets HS-HSPB interactions.
